# Chaotic Sparrow Search Algorithm with Deep Transfer Learning Enabled Breast Cancer Classification on Histopathological Images

**DOI:** 10.3390/cancers14112770

**Published:** 2022-06-02

**Authors:** K. Shankar, Ashit Kumar Dutta, Sachin Kumar, Gyanendra Prasad Joshi, Ill Chul Doo

**Affiliations:** 1 Big Data and Machine Learning Laboratory, South Ural State University, 454080 Chelyabinsk, Russia; drkshankar@ieee.org (K.S.); kumars@susu.ru (S.K.); 2 Department of Computer Science and Information System, College of Applied Sciences, AlMaarefa University, Riyadh 11597, Saudi Arabia; adotta@mcst.edu.sa; 3 Department of Computer Science and Engineering, Sejong University, Seoul 05006, Korea; 4 Artificial Intelligence Education, Hankuk University of Foreign Studies, Dongdaemun-gu, Seoul 02450, Korea

**Keywords:** breast cancer, histopathological images, computer aided diagnosis, cancer, medical imaging, deep learning

## Abstract

**Simple Summary:**

Cancer is considered the most significant public health issue which severely threatens people’s health. The occurrence and mortality rate of breast cancer have been growing consistently. Initial precise diagnostics act as primary factors in improving the endurance rate of patients. Even though there are several means to identify breast cancer, histopathological diagnosis is now considered the gold standard in the diagnosis of cancer. However, the difficulty of histopathological image and the rapid rise in workload render this process time-consuming, and the outcomes might be subjected to pathologists’ subjectivity. Hence, the development of a precise and automatic histopathological image analysis method is essential for the field. Recently, the deep learning method for breast cancer pathological image classification has made significant progress, which has become mainstream in this field. Therefore, in this work, we focused on the design of metaheuristics with deep learning based breast cancer classification process. The proposed model is found to be an effective tool to assist physicians in the decision making process.

**Abstract:**

Breast cancer is the major cause behind the death of women worldwide and is responsible for several deaths each year. Even though there are several means to identify breast cancer, histopathological diagnosis is now considered the gold standard in the diagnosis of cancer. However, the difficulty of histopathological image and the rapid rise in workload render this process time-consuming, and the outcomes might be subjected to pathologists’ subjectivity. Hence, the development of a precise and automatic histopathological image analysis method is essential for the field. Recently, the deep learning method for breast cancer pathological image classification has made significant progress, which has become mainstream in this field. This study introduces a novel chaotic sparrow search algorithm with a deep transfer learning-enabled breast cancer classification (CSSADTL-BCC) model on histopathological images. The presented CSSADTL-BCC model mainly focused on the recognition and classification of breast cancer. To accomplish this, the CSSADTL-BCC model primarily applies the Gaussian filtering (GF) approach to eradicate the occurrence of noise. In addition, a MixNet-based feature extraction model is employed to generate a useful set of feature vectors. Moreover, a stacked gated recurrent unit (SGRU) classification approach is exploited to allot class labels. Furthermore, CSSA is applied to optimally modify the hyperparameters involved in the SGRU model. None of the earlier works have utilized the hyperparameter-tuned SGRU model for breast cancer classification on HIs. The design of the CSSA for optimal hyperparameter tuning of the SGRU model demonstrates the novelty of the work. The performance validation of the CSSADTL-BCC model is tested by a benchmark dataset, and the results reported the superior execution of the CSSADTL-BCC model over recent state-of-the-art approaches.

## 1. Introduction

Cancer is considered the most significant public health issue which severely threatens people’s health. The occurrence and mortality rate of breast cancer (BC) have been growing consistently. Initial precise diagnostics act as primary factors in improving the endurance rate of patients [1]. A mammogram is the starting stage of initial prognosis; hence, it becomes hard to detect cancer in the denser breasts of teenage women. X-ray radiation warns radiologists of the patient’s health [2]. The golden standard for BC prognosis is only pathological examination. Pathological examinations generally attain tumor samples via excision, puncture, etc. [3]. Hematoxylin combines deoxyribonucleic acid (DNA), and eosin combines proteins. The precise prognosis of BC demands proficient histopathologists, and it needs more time and endeavor to finish this work. Moreover, the prognosis outcomes of distinct histopathologists are dissimilar and heavily based on histopathologists’ earlier experience [4].

Recently, BC prognosis is dependent on the histopathological image, and this is confronted by three major difficulties. At first, there is a shortcoming of proficient histopathologists across the globe, particularly in quite a few undeveloped regions and small hospitals [5]. Next, the prognosis of histopathologists is subjective, and evaluation is not performed on an objective basis. Whether prognosis is right or not is wholly based on the histopathologists’ earlier knowledge [6]. Lastly, the prognosis of BC depends on the histopathological image, which is time consuming, highly complex, and labor-intensive, and it is considered ineffective during the era of big data. Despite such issues, an objective and effective BC prognosis technique is essential for mitigating the pressure of the workload of histopathologists [7]. The speedy advancement of computer-aided diagnosis (CAD) was slowly employed in the clinical domain. The CAD system will not act as a substitute for the physician; however, it can be utilized as a “second reader” in assisting the physician in recognizing diseases [8]. However, there are false-positive areas identified by the computer that will consume time for the physician in evaluating the outcomes induced by the computer, again leading to a decline in effectiveness and preciseness. Thus, methods for improving the sensitiveness of computer-aided tumor identification methodologies while greatly minimizing the incorrect positive identification rate and enhancing the efficiency of the identification technique constitute a potential research area [9].

Currently, deep learning (DL) methods have become popular in computer vision (CV), particularly in biomedical image processing. These methods were able to investigate complex and enhanced characteristics from images automatically. At the same time, these methods greatly require the attention of several authors in using such techniques to categorize BC histopathology images [10]. In particular terms, convolutional neural networks (CNNs) are broadly utilized in image-based works because of their capabilities to efficiently distribute variables over several layers inside a DL method.

This study introduces a novel chaotic sparrow search algorithm with a deep transfer learning-enabled breast cancer classification (CSSADTL-BCC) model applied on histopathological images. The presented CSSADTL-BCC model applies the Gaussian filtering (GF) approach to eradicate the occurrence of noise. In addition, a MixNet-based feature extraction model was employed to generate a useful set of feature vectors. Furthermore, a CSSA with a stacked gated recurrent unit (SGRU) classification approach was exploited to allot class labels. The CSSADTL-BCC model does not exist in the literature to the best of our knowledge. The design of the CSSA for optimal hyperparameter tuning of the SGRU model demonstrates the novelty of the work. The performance validation of the CSSADTL-BCC model was verified using benchmark data collection, and the outcomes were inspected under different evaluation measures.

The remaining sections of the paper are planned as follows. Section 2 indicates the existing works related to BC classification. Next, Section 3 elaborates the proposed model, and Section 4 offers the performance validation. At last, Section 5 draws the conclusions.

## 2. Literature Review

In [11], the authors proposed a real time data augmentation-related transfer learning method to resolve existing limitations. Two popular and well-established image classification methods, such as Xception and InceptionV3 frameworks, have been trained on a freely accessible BC histopathological image data named BreakHis. Alom et al. [12] presented a technique for classifying BC using the Inception Recurrent Residual Convolution Neural Network (IRRCNN) framework. The proposed method is an effective DCNN system that integrates the strength of the Recurrent Convolution Neural Network (RCNN), Inception Network (Inception-v4), and the Residual Network (ResNet). The experiment result illustrates better performance against RCNN, Inception Network, and ResNet for object-detection tasks.

Vo et al. [13] presented a technique that employs the DL method with a convolution layer for extracting the visual feature for BC classification. It has been found that the DL model extracts the most useful feature when compared to the handcrafted feature extraction approach. In [14], the authors proposed a BC histopathological image categorization related to deep feature fusion and enhanced routing (FE-BkCapsNet) to exploit CapsNet and CNN models. Firstly, a new architecture with two channels could simultaneously extract capsule and convolutional features and incorporate spatial and sematic features into the new capsule to obtain a discriminative dataset.

The researchers in [15] proposed a patch-based DL method named Pa-DBN-BC for classifying and detecting BC on histopathology images with the Deep Belief Network (DBN). The feature is extracted by supervised finetuning and unsupervised pre-training phases. The network extracts feature automatically from image patches. Logistic regression is utilized for classifying the patches from histopathology images. In [16], the authors proposed a robust and novel technique based convolution-LSTM (CLSTM) learning method, the pre-processing method with the optimized SVM classifier, and the marker-controlled watershed segmentation algorithm (MWSA) for automatically identifying BC. Saxena et al. [17] presented a hybrid ML method for solving class imbalance problems. The presented method uses the kernelized weighted ELM and pre-trained ResNet50 for CAD of BC using histopathology.

Several automated breast cancer classification models are available in the literature. However, the models still contains a challenging problem. Because of the continual deepening of models, the number of parameters of DL models also increases quickly, which results in model overfitting. At the same time, different hyperparameters have a significant impact on the efficiency of the CNN model, particularly in terms of the learning rate. Modifying the learning rate parameter for obtaining better performance is also required. Therefore, in this study, we employ the CSSA technique for the hyperparameter tuning of the SGRU model.

## 3. The Proposed Model

**Figure 1:**
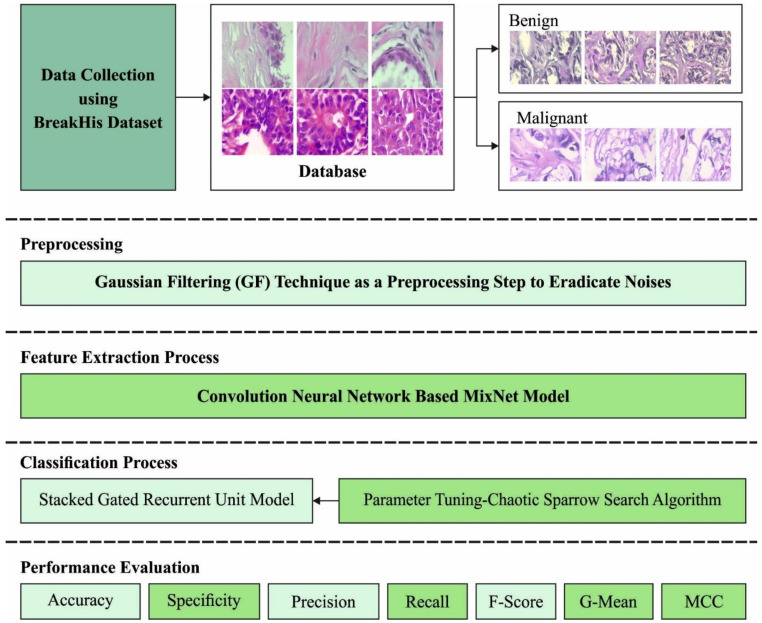
The overall process of the CSSADTL-BCC technique.

In this study, a new CSSADTL-BCC model was developed to classify BC on histopathological images. The presented CSSADTL-BCC model mainly focused on the recognition and classification of BC. At the primary stage, the CSSADTL-BCC model employed the GF technique to eradicate the occurrence of noise. It was then followed by using a MixNet-based feature extraction model employed to produce a useful set of feature vectors. Then, the CSSA-SGRU classifier was exploited to allot class labels. Figure 1 illustrates the overall process of the CSSADTL-BCC technique.

### 3.1. Image Pre-Processing

At the primary stage, the CSSADTL-BCC model employed the GF technique to eradicate the occurrence of noise. GF is a bandpass filter, viz., efficiently implemented in machine vision and image processing applications [18]. A two-dimensional Gabor purpose was oriented by sinusoidal grates controlled by two dimensional Gaussian envelopes. In the two-dimensional coordinate $\left(a,b\right)$ model, the GF comprising an imaginary and real one is illustrated by the following:

(1) $$G_{\delta ,\theta ,\psi ,\sigma ,\gamma }\left(a,b\right)=\exp\left(-\frac{{a^{\prime }}^{2}+\gamma^{2}{b^{\prime }}^{2}}{2\sigma^{2}}\right)\times \exp\left(j\left(2\pi \frac{a^{\prime }}{\delta }+\psi \right)\right)$$

where they are described as follows.

(2) $$a^{\prime }=a\;\cos\;\theta +b\;\sin\;\theta$$

(3) $$b^{\prime }=-a\;\sin\;\theta +b\;\cos\;\theta$$

Now θ implies the orientation separation angle of the Gabor kernel, and δ signifies the wavelength of sinusoidal features. Notably, it is essential to consider θ from the range $\left[0^{o},\;180^{o}\right]$ as symmetry generates another redundant direction. ψ denotes the stage offset, σ indicates the standard derivation of the Gaussian envelope, and γ represents the ratio of spatial features for identifying the ellipticity of the Gabor role. ψ=0 and ψ=π/2 return the real and imaginary parts of GF. Variable 0 can be determined as 6 and spatial frequency bandwidth bw is given by the following.

(4) $$\sigma =\frac{\delta }{pi}\sqrt{\frac{ln2}{2}\;\;}\frac{2^{bw}+1}{2^{bw}-1}$$

### 3.2. MixNet-Based Feature Extractor

Next, for image pre-processing, a MixNet-based feature extraction model is employed to generate a useful set of feature vectors. A CNN algorithm created by the traditional convolutional operation is difficult to use for mobile terminals due to its complicated calculations and excessive parameters. In order to improve its effectiveness on mobile terminals and to guarantee the accuracy of the model, a sequence of lightweight convolutional operators has been presented. Amongst them, one of the most commonly utilized is a depthwise separable convolution layer. A depthwise separable convolutional layer splits the convolution into pointwise and depthwise convolution. In the initial phase, it convolves a single channel at a time using convolutional kernels at size = 3. In the second phase, it uses a feature map with the 1 × 1 convolutional kernel. Assume that *N* $D_{k}\times D_{k}$ feature view and 1 convolutional sliding step are utilized to convolve a feature map with $D_{F}\times D_{F}\times M$ dimensions, including the output feature map with dimensions of $D_{F}\times D_{F}\times N$. The parameter amount of traditional convolutional operations is provided as follows.

(5) $$D_{k}\times D_{k}\times M\times N$$

The parameters involved in the depthwise separable convolutional operation is provided below.

(6) $$D_{k}\times D_{k}\times M+1\times 1\times M\times N\;$$

The computation involved in traditional convolutional operation is provided as follows.

(7) $$D_{k}\times D_{k}\times M\times N\times D_{F}\times D_{F}$$

The computation involved in depthwise separable convolutional operation is defined in Equation (8).

(8) $$D_{k}\times D_{k}\times M\times D_{F}\times D_{F}\times M\times N\times D_{F}\times D_{F}$$

The ratio of the two operations is provided as follows.

(9) $$\frac{D_{k}\times D_{k}\times M\times D_{F}\times D_{F}\times M\times N\times D_{F}\times D_{F}}{D_{k}\times D_{k}\times M\times N\times D_{F}\times D_{F}}$$

A depthwise separable convolutional layer uses a similar size 3 × 3 convolutional kernel in the computation method; however, a network with larger convolutional kernels of 5 × 5 or 7 × 7 confirms that a larger convolutional kernel improves the efficiency and accuracy of the model. However, the experiment shows that the case where a larger convolutional kernel is better is rare; simultaneously, a large convolutional kernel minimizes the model’s accuracy. Here, MDConv splits the input channel with *M* size into C groups, later convolving all the groups with distinct kernel sizes. The standard depthwise separable convolution splits the input channel with *M* size into *M* groups and later implements convolutional calculations for all groups with a similar kernel size.

### 3.3. Image Classification Using SGRU Model

**Figure 2:**
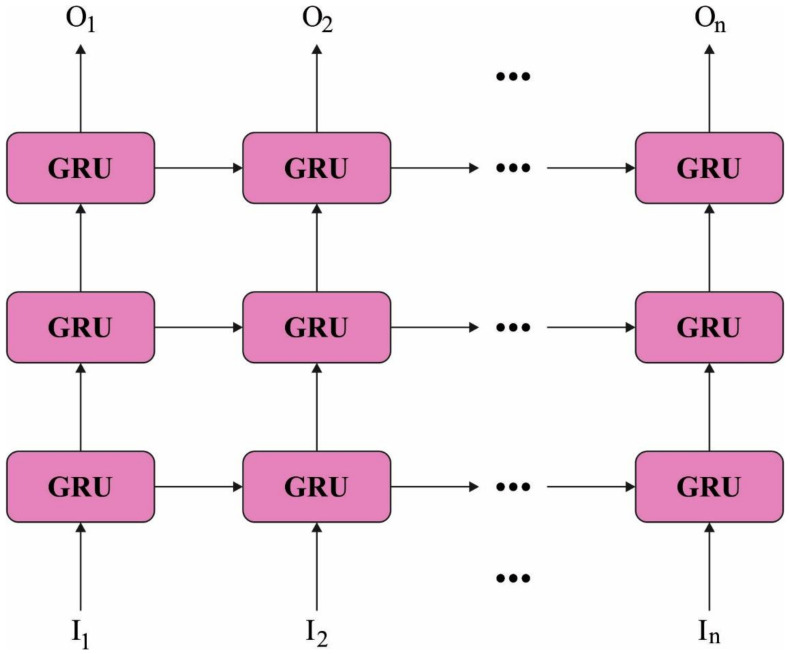
Framework of SGRU model.

At this stage, the generated feature vectors are passed into the SGRU classifier to allot class labels. SGRU is made up of various GRU units. For time series t, the input series $\left\{e_{1},\;e_{2},\;\ldots ,\;e_{t}\right\}$ first enters into hidden layer $\left\{h_{1}^{1},\;h_{2}^{1},\;\ldots ,\;h_{t}^{1}\right\}$ to attain all data from the previous time step. Next, the upper hidden layer takes the output from the lower hidden layers at a similar time step as the input for extracting features [19]. In particular, the upper layer of the hidden layer is $\left\{h_{1}^{2},\;h_{2}^{2},\;\ldots ,\;h_{t}^{2}\right\}.$ For all layers, a hidden layer $h_{t}^{i}$, as provided in Equation (13), is shown by Equations (10)–(12) to attain the candidate value, update, and reset gates. It should be noted that in Equations (10)–(12), we have included embedding vector $e_{t}$ in the initial layer. Starting from the next layer upward, we employ the hidden state from the current time step in the previous layer, $h_{t}^{i-1}$, rather than $e_{t}$ in (10)–(12). Figure 2 depicts the framework of SGRU.

(10) $$u_{t}^{i}=\sigma \left(W_{u}^{i}h_{t-1}^{i}+U_{u}^{i}e_{t}+b_{u}^{i}\right)$$

(11) $$r_{t}^{i}=\sigma \left(W_{r}^{i}h_{t-1}^{i}+U_{r}^{i}e_{t}+b_{r}^{i}\right)$$

(12) $$\tilde{C}=\tanh\left(W_{c}^{i}.\left[r_{t}^{i}\times h_{t-1}^{i}\right]+U_{c}^{i}e_{t}+b_{c}^{i}\right)$$

(13) $$h_{t}^{i}=u_{t}^{i}\times {\tilde{C}}_{t}^{i}+\left(1-u_{t}^{i}\right)\times h_{t-1}^{i}$$

### 3.4. Hyperparameter Optimization

Finally, CSSA is implied to optimally modify the hyperparameters included in the MixNet model. SSA attains the best possible solution by mimicking certain behaviors of sparrows [20]. Firstly, the discoverer–joiner sparrow population models are established, and then the sparrow is arbitrarily chosen as a guard. The joiner snatches food from the discoverer, observes the discoverer, and follows the discoverer for food. The discoverer takes the responsibility to provide foraging direction and areas for the sparrow population. Once the vigilante realizes the threat, the population implements anti-predation behavior immediately. Lastly, with various iterations of the location of the discoverer and joiner, the adoptive position for the entire population can be found. The sparrow population is within the space of N×D, where N indicates the overall amount of sparrows, D represents the spatial dimension. Next, the location of the *i*-th sparrow in space represents $X_{i}=\left(x_{i1},\;x_{i2},\;\cdots ,\;x_{id}\right)$, $i\in \left[1,\;N\right],$ $d\in \left[1,\;D\right],$ and $x_{id}$ characterizes the location of *i*-th sparrow in d-dimension. The position update equation of the discoverer can be shown in the following Equation (14).

(14) $$x_{id}^{t+1}=\left\{\begin{array}{cc} x_{id}^{t}\cdot exp.\left(\frac{-i}{\alpha \cdot T}\right) & R_{2}<ST\\ x_{id}^{t}+Q\cdot L, & R_{2}\geq ST \end{array}\right.$$

In the equation, t signifies the existing amount of iterations; T indicates the maximal amount of iterations; α represents an arbitrary value within [0, 1];Q implies an arbitrary value with standard distribution; L indicates a matrix in that element is 1, and its size is 1×d; $R_{2}\in \left[0,1\right]$ signifies the warning values; $ST\in \left[0.5,1\right]$ denotes the safety values. If $R_{2}<ST$, this implies that the population is not at risk and the discoverer continues searching. If $R_{2}\geq$ ST, this implies that the vigilante discovered the predator and instantly delivered an alarm to the others. The sparrow population implements anti-predation behavior immediately any fly to a safer region for food. The position update equation of the joiner can be shown in the following Equation (15).

(15) $$x_{id}^{t+1}=\left\{\begin{array}{cc} Q\cdot exp\left(\frac{x_{worstd}^{t}-x_{id}^{t}}{i^{2}}\right) & i>\frac{N}{2}\\ x_{best\;d}^{t+1}+\frac{1}{D}\overset{D}{\underset{d=1}{\sum }}(rand\left(-1,1\right)\cdot |x_{id}^{t}-x_{best\;d}^{t+1} & i\leq \frac{N}{2} \end{array}\right.$$

Here, $x_{worstd}^{t}$ signifies the global worst place in tth iteration; $x_{bestd}^{t+1}$ signifies the global optimal location at the tth iteration. If $i>\frac{N}{2}$, it implies that the *i*-th joiner has not attained food and that it needs to fly toward another location in order to search for food. If $i\leq \frac{N}{2},$ this implies that the *i*-th joiner is closer to the world’s best location and is arbitrarily foraging around. The vigilant location upgrade equation is provided as follows:

(16) $$x_{id}^{r+1}=\left\{\begin{array}{cc} x_{worst\;d}^{t}+\beta \left(x_{id}^{t}-x_{worst\;d}^{t}\right), & f_{i}\neq f_{g}\\ x_{id}^{t}+K\left(\frac{x_{id}^{t}-x_{worst\;d}^{t}}{\left|f_{i}-f_{w}\right|+e}\right) & f_{i}=f_{g} \end{array}\right.$$

where β signifies the step length control variable that is an arbitrary value subjected to a regular distribution with a variance of 1 and means value of 0; K denotes the movement direction of sparrow, and arbitrary values lie within [1, 1]; e indicates a constant with smaller value; $f_{i}$ characterizes the fitness of *i*-th sparrow; $f_{g}$ signifies the optimum fitness of the existing population; $f_{w}$ denotes the worst fitness of existing population. If $f_{i}\neq f_{g}$, this implies that the *i*-th sparrow is at the edge of the population and can be attacked easily by the predator. If $f_{i}=f_{g}$, this implies that *i*-th sparrow is within center of the population, and it is aware of danger; it relocates closer to other sparrows in order to reduce the threat of becoming caught.

With the addition of a global optimum sparrow neighborhood in all iterations, the searching ability of SSA can be enhanced. Additionally, this could assist the sparrow group in attaining the best location through the search process. The chaotic local searching technique can be employed in the iteration process of SSA for improving the capability of exploitation and maintaining a better harmony among the core search processes. Moreover, the logical chaotic function is employed to calculate chaotic SSA. This can be obtained as follows.

(17) $$\rho_{k+1}=\mu \rho_{k}\left(1-\rho_{k}\right),\;k=1,2,\;\ldots ,\;N-1$$

On the other hand, $\rho_{1}\in \left(0,1\right)$ and $\rho_{1}\neq 0.25,\;0.5,\;0.75$, and 1 once the control parameter μ is set to 4, and the logistic function is converted to a chaotic state. Therefore, the chaotic local searching function is shown below.

(18) $$P_{i}=b+\rho_{i}\times \left(b-a\right),\;i=1,2,\ldots ,N$$

Here, $\left[a,\;b\right]$ indicates the searching space, and the chaotic function was produced by mapping chaotic parameters $\rho_{i}$ into the chaotic vector $P_{i}$. Furthermore, chaotic vector $P_{i}$ was linearly integrated with targeted position TP for generating candidate location CL, which is expressed as follows.

(19) $$CL=\left(1-SC\right)\times TP+SC\times P_{i}$$

(20) $$SC=\left(T-t+1\right)/T$$

The CSSA approach resolves an FF for obtaining higher classification performances. It defines a positive integer for demonstrating the optimal performance of candidate solutions. During this case, the minimized classifier error rate was regarded as FF, as offered in Equation (21).

(21) $$fitness\left(x_{i}\right)=ClassifierErrorRate\left(x_{i}\right)\;\;\;\;\;\;\;\;\;\;\;\;\;\;=\frac{number\;of\;misclassified\;samples}{Total\;number\;of\;samples}\times 100$$

## 4. Performance Validation

**Figure 3:**
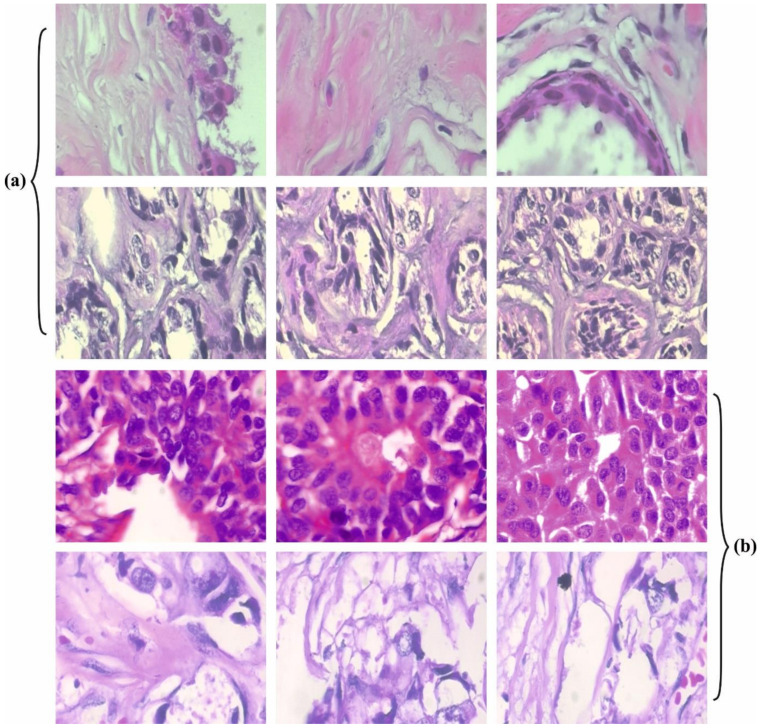
Sample images: (**a**) benign (**b**) malignant.

In this section, the experimental validation of the CSSADTL-BCC model is tested using a benchmark dataset [21], and the details are provided in Table 1. The CSSADTL-BCC model is simulated using the Python 3.6.5 tool. The parameter settings are provided as follows: learning rate—0.01; dropout—0.5; batch size—5; epoch count—50; activation—ReLU. A few sample images are demonstrated in Figure 3.

**Figure 4:**
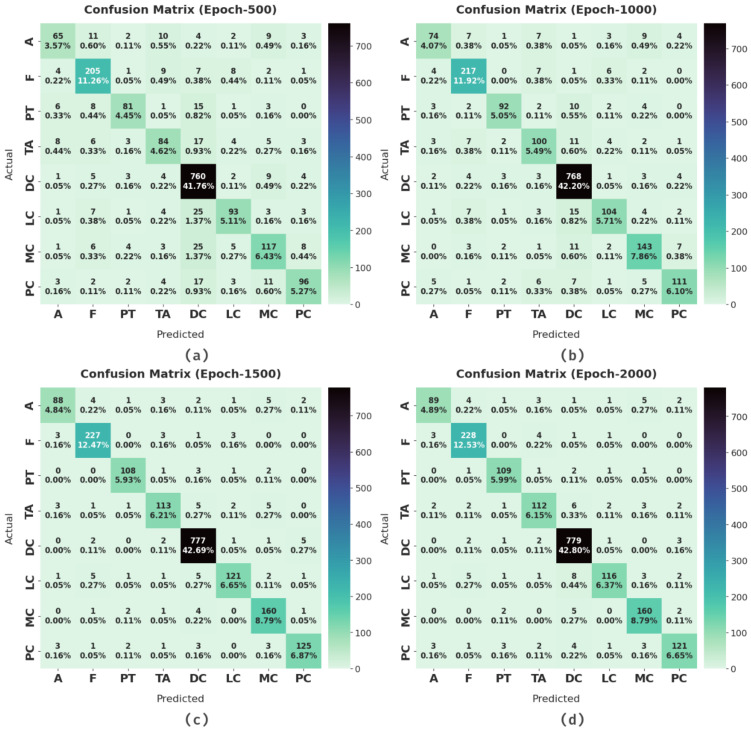
Confusion matrix of CSSADTL-BCC technique under various epochs: (**a**) 500 epochs, (**b**) 1000 epochs, (**c**) 1500 epochs, and (**d**) 2000 epochs.

Figure 4 illustrates the confusion matrices produced by the CSSADTL-BCC model under distinct epochs. With 500 epochs, the CSSADTL-BCC model has identified 65 samples in class A, 205 samples in class F, 81 samples in class PT, 84 samples in class TA, 760 samples in class DC, 93 samples in class LC, 117 samples in class MC, and 96 samples in class PC. Along with that, with 2000 epochs, the CSSADTL-BCC approach has identified 89 samples in class A, 228 samples in class F, 109 samples in class PT, 112 samples in class TA, 779 samples in class DC, 116 samples in class LC, 160 samples in class MC, and 121 samples in class PC.

**Figure 5:**
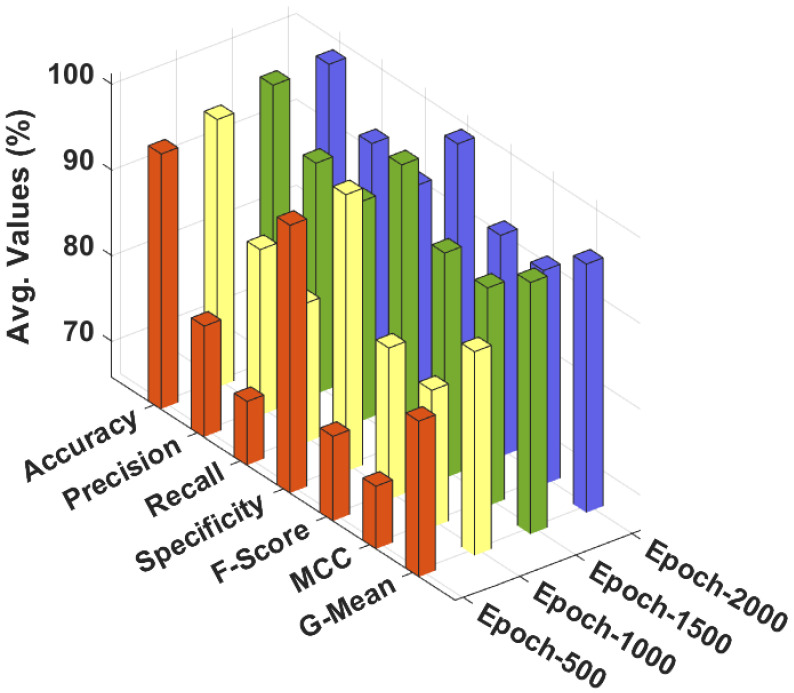
Result analysis of CSSADTL-BCC technique with distinct epochs.

Table 2 and Figure 5 highlight the overall classification outcomes of the CSSADTL-BCC model under distinct epochs and class labels. The experimental outcomes implied that the CSSADTL-BCC model has resulted in ineffectual outcomes over other models in terms of different measures such as accuracy ($accu_{y}$), precision ($prec_{n}$), recall ($reca_{l}$), specificity ($spec_{y}$), F-score ($F_{score}$), MCC, and G-mean ($G_{mean}$). For instance, with 500 epochs, the CSSADTL-BCC model provided the averages of $accu_{y}$, $prec_{n}$, $reca_{l}$, $spec_{y}$, $F_{score}$, MCC, and $G_{mean}$ at 95.62%, 78.78%, 73.25%, 97.09%, 75.71%, 73.18%, and 84.01%, respectively. Moreover, with 1000 epochs, the CSSADTL-BCC method obtained the averages of $accu_{y}$, $prec_{n}$, $reca_{l}$, $spec_{y}$, $F_{score}$, MCC, and $G_{mean}$ at 97.10%, 85.21%, 82.09%, 98.16%, 83.52%, 81.84%, and 89.62%, respectively. In addition, with 1500 epochs, the CSSADTL-BCC methodology provided averages of $accu_{y}$, $prec_{n}$, $reca_{l}$, $spec_{y}$, $F_{score}$, MCC, and $G_{mean}$ at 98.61%, 92.80%, 91.48%, 99.14%, 92.10%, 91.29%, and 95.19%, respectively. At last, with 2000 epochs, the CSSADTL-BCC technique obtained the averages of $accu_{y}$, $prec_{n}$, $reca_{l}$, $spec_{y}$, $F_{score}$, MCC, and $G_{mean}$ at 98.54%, 92.58%, 90.87%, 99.08%, 91.66%, 90.82%, and 94.84%, respectively.

**Figure 6:**
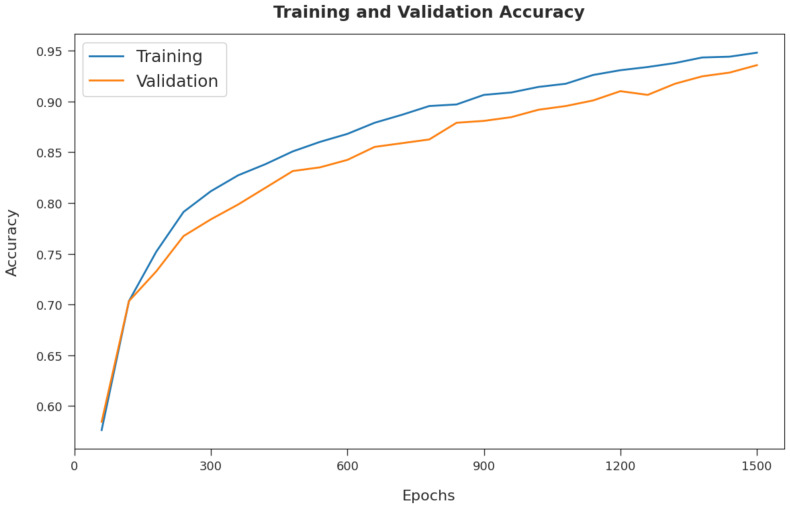
TA and VA analysis of CSSADTL-BCC technique.

The training accuracy (TA) and validation accuracy (VA) attained by the CSSADTL-BCC model on test dataset are demonstrated in Figure 6. The experimental outcomes implied that the CSSADTL-BCC model has gained maximum values of TA and VA. In particular, VA appeared to be higher than TA.

**Figure 7:**
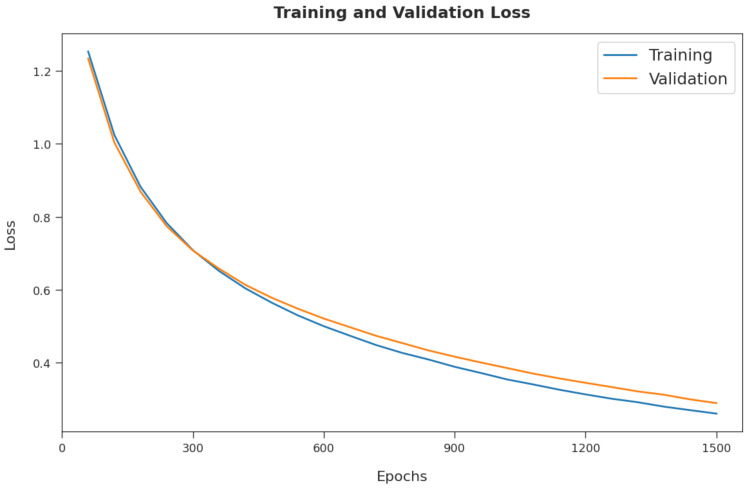
TL and VL analysis of CSSADTL-BCC technique.

**Figure 8:**
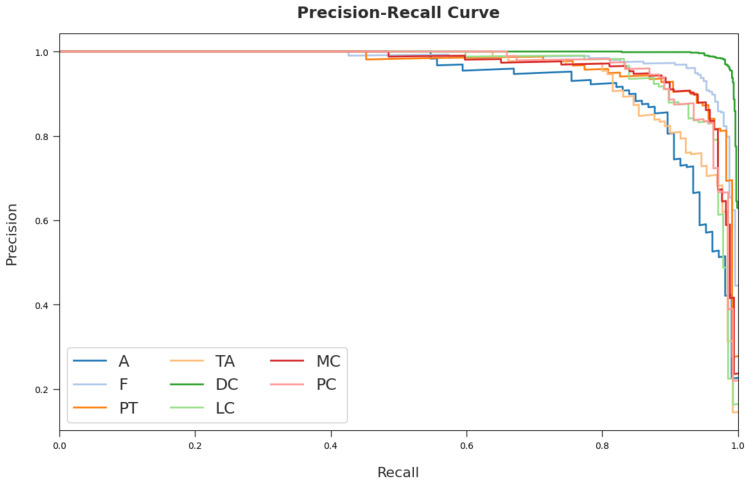
Precision–recall curve analysis of CSSADTL-BCC technique.

The training loss (TL) and validation loss (VL) achieved by the CSSADTL-BCC method on test dataset are established in Figure 7. The experimental outcome inferred that the CSSADTL-BCC model obtained the lowest values of TL and VL. In particular, VL seemed to be lower than TL. Next, a brief precision–recall examination performed on the CSSADTL-BCC method on the test dataset is displayed in Figure 8. By observing the figure, it can be observed that the CSSADTL-BCC approach has established maximal precision–recall performance under all classes.

**Figure 9:**
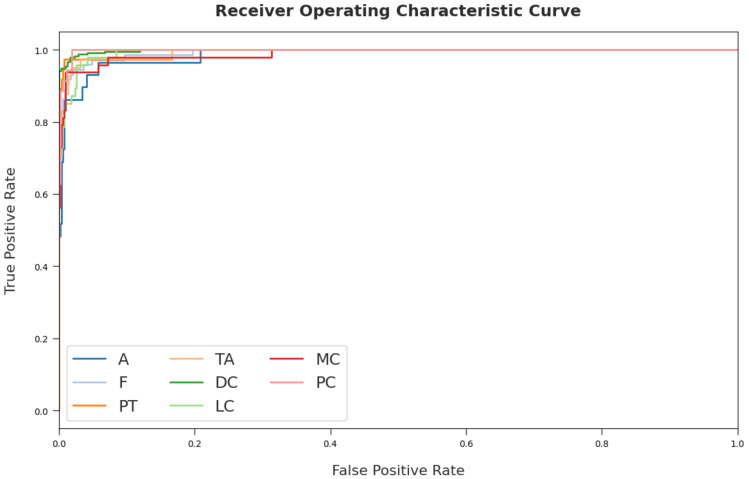
ROC curve analysis of the CSSADTL-BCC technique.

Figure 9 portrays a clear ROC investigation of the CSSADTL-BCC model on the test dataset. The figure portrayed that the CSSADTL-BCC model has resulted in proficient results with maximum ROC values under distinct class labels.

**Figure 10:**
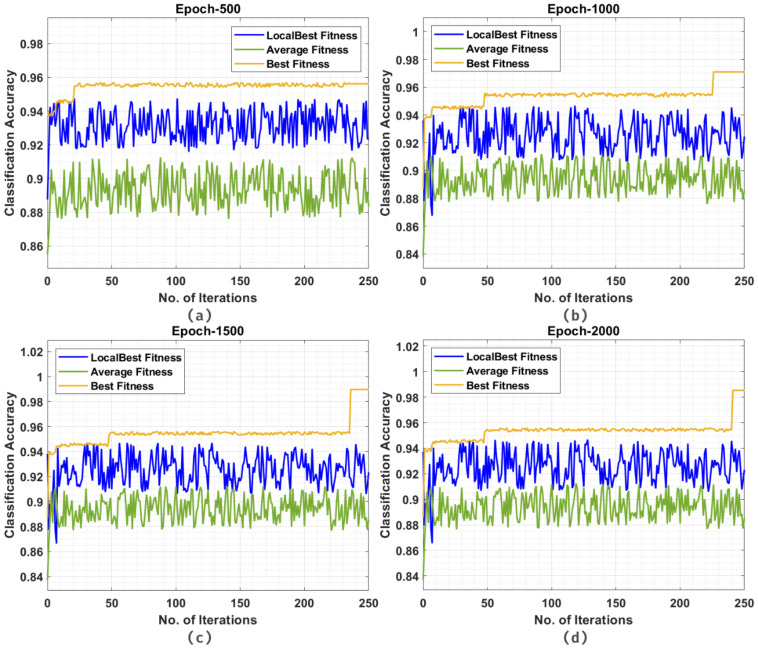
Classification accuracy of CSSADTL-BCC technique under distinct iterations: (**a**) 500 epochs, (**b**) 1000 epochs, (**c**) 1500 epochs, and (**d**) 2000 epochs.

Figure 10 reports detailed classification accuracy outcomes of the CSSADTL-BCC model under distinct iterations and runs. The figures highlighted that CSSADTL-BCC has showcased effectual classifier results under every epoch.

**Figure 11:**
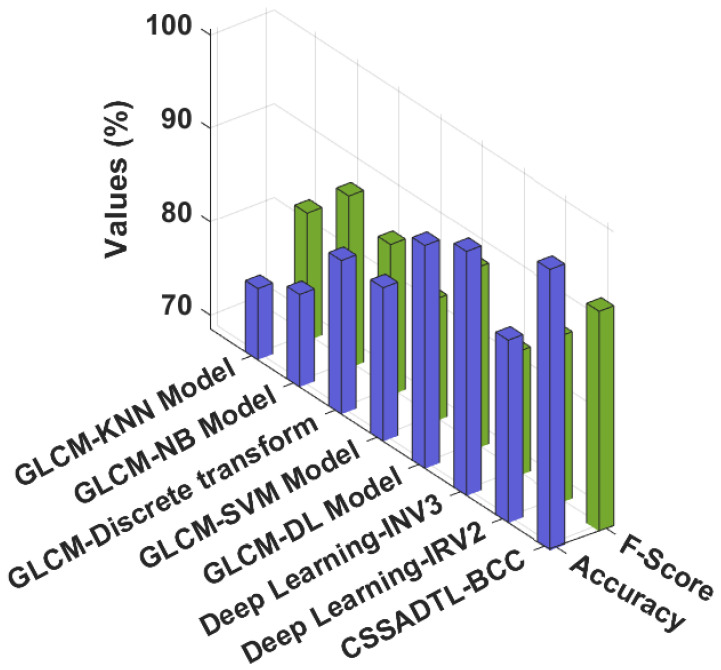
$Accu_{y}$ and $F_{score}$ analysis of CSSADTL-BCC technique with existing algorithms.

To highlight the enhanced outcomes of the CSSADTL-BCC model, a brief comparison study with recent models is shown in Table 3 [22]. Figure 11 investigates a detailed $accu_{y}$ and $F_{score}$ analysis of the CSSADTL-BCC with existing models. The results indicated that GLCM-KNN and GLCM-NB models obtained lower values of $accu_{y}$ and $F_{score}.$ At the same time, the GLCM-discrete transform, GLCM-SVM, and Deep learning-IRV2 models have attained moderately closer values of $accu_{y}$ and $F_{score}$. Next to that, the GLCM-DL and Deep learning INV3 models have resulted in reasonable $accu_{y}$ and $F_{score}$ values. However, the CSSADTL-BCC model has gained an effectual outcome with maximum $accu_{y}$ and $F_{score}$ at 98.61% and 92.80%, respectively.

**Figure 12:**
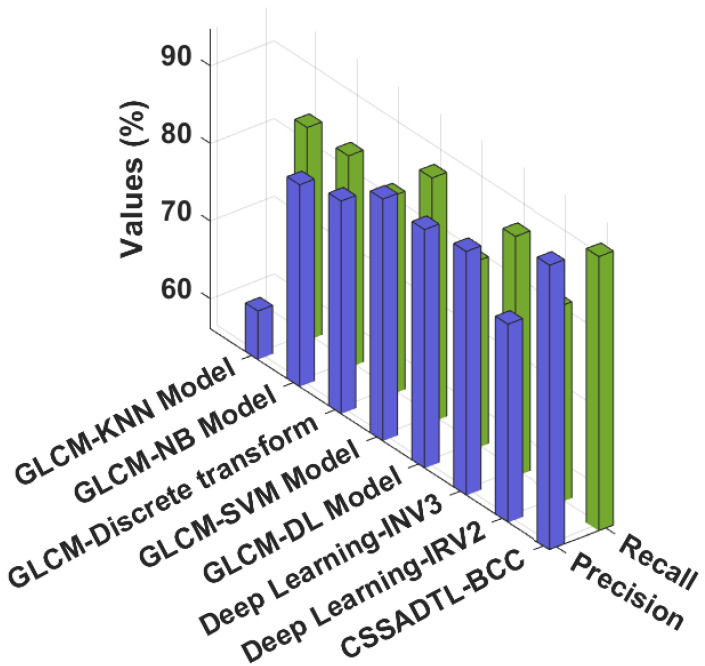
$Reca_{l}$ and $Prec_{n}$ analysis of the CSSADTL-BCC technique with existing algorithms.

Figure 12 examines a detailed $prec_{n}$ and $reca_{l}$ examination of CSSADTL-BCC with existing techniques. The outcomes represented that the GLCM-KNN and GLCM-NB approaches have gained lesser values of $prec_{n}$ and $reca_{l}.$ Moreover, the GLCM-discrete transform, GLCM-SVM, and Deep learning-IRV2 algorithms have attained moderately closer values of $prec_{n}$ and $reca_{l}$. Along with that, the GLCM-DL and Deep learning INV3 approaches have resulted in reasonable $prec_{n}$ and $reca_{l}$ values. However, the CSSADTL-BCC technique has gained effectual outcomes with maximum values of $prec_{n}$ and $reca_{l}$ at 92.80% and 91.48%, respectively. After observing the results and discussion, it is apparent that the CSSADTL-BCC model has showcased enhanced outcomes over other methods. The enhanced performance of the CSSADTL-BCC model is due to the effectual hyperparameter tuning process of the SGRU classifier. Thus, the proposed model can be applied to assist physicians in the disease diagnosis process.

## 5. Conclusions

In this study, a new CSSADTL-BCC method was advanced for classifying BC on histopathological images. The presented CSSADTL-BCC model mainly focused on the recognition and classification of BC. At the primary stage, the CSSADTL-BCC model employed the GF technique to eradicate the occurrence of noise. Moreover, a MixNet-based feature extraction model was employed for producing a useful collection of feature vectors. Then, the SGRU classifier was exploited to allot class labels. Furthermore, CSSA is applied to optimally modify the hyperparameters involved in the MixNet model. The performance validation of the CSSADTL-BCC model can be tested by using a benchmark dataset, and the results reported the superior efficiency of the CSSADTL-BCC method over the current existing approaches with a maximum accuracy of 98.61%. In the future, deep instance segmentation approaches can be included to enhance classification performance. In addition, the classifier’s results can be boosted by designing deep fusion-based ensemble models.

## Figures and Tables

**Table 1:** Dataset details.

Category	Class Names	Labels	No. of Images	Total
Benign	Adenosis	A	106	588
	Fibroadenoma	F	237	
	Phyllodes Tumor	PT	115	
	Tubular Adenoma	TA	130	
Malignant	Carcinoma	DC	788	1232
	Lobular Carcinoma	LC	137	
	Mucinous Carcinoma	MC	169	
	Papillary Carcinoma	PC	138	
Total Number of Images				1820

**Table 2:** Result analysis of CSSADTL-BCC technique with various measures and epochs.

Class Labels	Accuracy	Precision	Recall	Specificity	F-Score	MCC	G-Mean
Epoch-500							
A	96.43	73.03	61.32	98.60	66.67	65.07	77.76
F	95.77	82.00	86.50	97.16	84.19	81.79	91.67
PT	97.25	83.51	70.43	99.06	76.42	75.27	83.53
TA	95.55	70.59	64.62	97.93	67.47	65.16	79.55
DC	92.42	87.36	96.45	89.34	91.68	85.10	92.83
LC	96.21	78.81	67.88	98.51	72.94	71.14	81.78
MC	94.84	73.58	69.23	97.46	71.34	68.54	82.14
PC	96.48	81.36	69.57	98.69	75.00	73.38	82.86
Average	95.62	78.78	73.25	97.09	75.71	73.18	84.01
Epoch-1000							
A	97.25	80.43	69.81	98.95	74.75	73.51	83.11
F	97.20	87.50	91.56	98.04	89.48	87.90	94.75
PT	98.13	89.32	80.00	99.35	84.40	83.56	89.15
TA	96.76	77.52	76.92	98.28	77.22	75.48	86.95
DC	95.82	93.20	97.46	94.57	95.29	91.61	96.01
LC	97.14	84.55	75.91	98.87	80.00	78.60	86.63
MC	96.98	83.14	84.62	98.24	83.87	82.21	91.18
PC	97.53	86.05	80.43	98.93	83.15	81.87	89.20
Average	97.10	85.21	82.09	98.16	83.52	81.84	89.62
Epoch-1500							
A	98.46	89.80	83.02	99.42	86.27	85.53	90.85
F	98.68	94.19	95.78	99.12	94.98	94.22	97.43
PT	99.23	93.91	93.91	99.59	93.91	93.50	96.71
TA	98.41	90.40	86.92	99.29	88.63	87.79	92.90
DC	98.13	97.12	98.60	97.77	97.86	96.21	98.19
LC	98.68	93.80	88.32	99.52	90.98	90.31	93.76
MC	98.52	89.89	94.67	98.91	92.22	91.44	96.77
PC	98.79	93.28	90.58	99.46	91.91	91.27	94.92
Average	98.61	92.80	91.48	99.14	92.10	91.29	95.19
Epoch-2000							
A	98.57	90.82	83.96	99.47	87.25	86.57	91.39
F	98.68	93.83	96.20	99.05	95.00	94.25	97.62
PT	99.18	92.37	94.78	99.47	93.56	93.13	97.10
TA	98.30	89.60	86.15	99.23	87.84	86.95	92.46
DC	98.02	96.65	98.86	97.38	97.74	96.00	98.12
LC	98.46	94.31	84.67	99.58	89.23	88.55	91.83
MC	98.68	91.43	94.67	99.09	93.02	92.31	96.86
PC	98.46	91.67	87.68	99.35	89.63	88.82	93.33
Average	98.54	92.58	90.87	99.08	91.66	90.82	94.84

**Table 3:** Comparative analysis of the CSSADTL-BCC technique with existing algorithms.

Methods	Accuracy	Precision	Recall	F-Score
GLCM-KNN Model	76.17	62.40	83.60	82.22
GLCM-NB Model	78.45	82.16	83.45	86.97
GLCM-Discrete transform	85.00	83.56	81.66	84.69
GLCM-SVM Model	85.00	87.32	87.61	81.62
GLCM-DL Model	92.44	86.89	80.24	87.92
Deep Learning-INV3	94.71	87.57	87.07	81.86
Deep Learning-IRV2	88.12	81.70	81.44	86.42
CSSADTL-BCC	98.61	92.80	91.48	92.10

## Data Availability

Data sharing not applicable to this article as no datasets were generated during the current study.
